# Supplementary material for: Screening of Macadamia integrifolia Varieties Based on the Comparison of Seedling Adaptability and Quality Differences
Source: Biology (Basel). 2025 Nov 21;14(12):1638. doi: 10.3390/biology14121638 (PMC12729574; doi:10.3390/biology14121638)
Supplement: Supplementary file 1 [file biology-14-01638-s001.zip › biology-3993933-supplementary.pdf]

Supplementary Figure legends

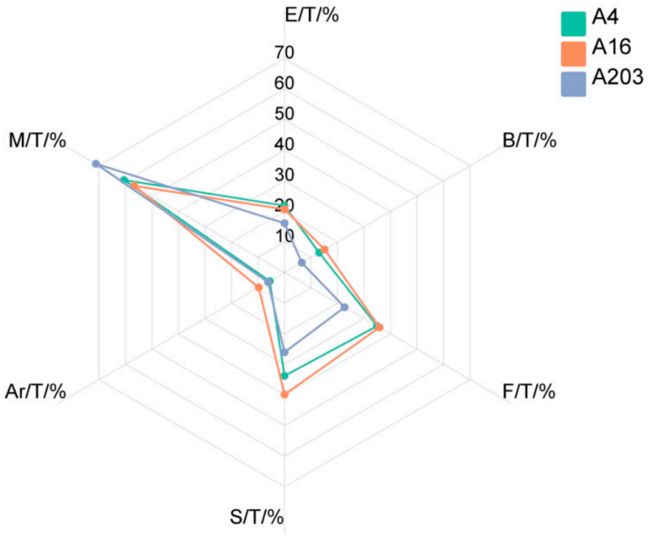

Figure S1 Classification of free amino acids in *Macadamia* nuts. E, Total essential amino acids; M, Total medicinal amino acids; S, Total sweet amino acids; F, Total flavor amino acids; B, Total bitter amino acids; Ar, Aromatic amino acids; T, Total amino acids.
